# Focus prediction of medical microscopic images based on Lightweight Densely Connected with Squeeze-and-Excitation Network

**DOI:** 10.3389/fnins.2023.1213176

**Published:** 2023-06-29

**Authors:** Hesong Jiang, Li Ma, Xueyuan Wang, Juan Zhang, Yueyue Liu, Dan Wang, Peihong Wu, Wanfen Han

**Affiliations:** ^1^School of Information Engineering, Southwest University of Science and Technology, Mianyang, China; ^2^The School of Internet of Things Engineering, Institute of Automation, Jiangnan University, Wuxi, China

**Keywords:** focus prediction, deep learning, medical microscopy, DenseNet, squeeze and excitation

## Abstract

Due to the demand for sample observation, optical microscopy has become an essential tool in the fields of biology and medicine. In addition, it is impossible to maintain the living sample in focus over long-time observation. Rapid focus prediction which involves moving a microscope stage along a vertical axis to find an optimal focus position, is a critical step for high-quality microscopic imaging of specimens. Current focus prediction algorithms, which are time-consuming, cannot support high frame rate imaging of dynamic living samples, and may introduce phototoxicity and photobleaching on the samples. In this paper, we propose Lightweight Densely Connected with Squeeze-and-Excitation Network (LDSE-NET). The results of the focusing algorithm are demonstrated on a public dataset and a self-built dataset. A complete evaluation system was constructed to compare and analyze the effectiveness of LDSE-NET, BotNet, and ResNet50 models in multi-region and multi-multiplier prediction. Experimental results show that LDSE-NET is reduced to 1E-05 of the root mean square error. The accuracy of the predicted focal length of the image is increased by 1 ~ 2 times. Training time is reduced by 33.3%. Moreover, the volume of the model only reaches the KB level, which has the characteristics of being lightweight.

## 1. Introduction

Nowadays, microscopy is still the most frequently used microscopic detection technology for examining thin sections and stained tissue sections on slides, playing an irreplaceable role in biomedicine, materials chemistry, industrial inspection, and other aspects (Ikeda et al., 2009; Zhang et al., 2014; Carrera et al., 2017). When the microscope is used for imaging living cells, defocus blur may occur due to thermal fluctuation of the microscope body and the movement of the microscope sample (Kreft et al., 2005). In addition, motion blur will also occur due to the uneven morphology of samples (Xu and Jia, 2010). Defocus blur and motion blur, as two of the most common microscopic imaging artifacts, can seriously degrade the imaging quality of digital pathology instruments (Redondo et al., 2012). Thus, maintaining the internal focal position of the microscope is a challenge. And when faced with a large number of samples, a large sample area, and a long observation time, manual focusing is impractical (Wang et al., 2018; Pinkard et al., 2019a). Therefore, autofocusing is crucial for high-precision microscope imaging.

The earliest research on autofocusing technique can be traced back to 1898 (Haosheng and Yu, 2021), but it was not until the 1960s that autofocusing technique was first used in the photographic system (Qiumei, 2006). Traditional autofocusing techniques almost use active focusing methods based on range finding, through the sensor to measure the distance to achieve (Lichang, 2015; Chen et al., 2020). With the gradual development of precision instruments toward intelligence and automation, higher requirements have been put forward for microscopes (Meng et al., 2022). Hence, a micro autofocusing technique based on digital image processing has gradually gained the attention of researchers (Kui, 2018). Image processing-based autofocusing methods are mainly divided into depth from defocus and depth from focus (Yunhao, 2019).

Depth from defocus was first proposed by Pentland in 1987, to obtain depth information from the defocused images and use optical principles to calculate the focal distance, to achieve the purpose of autofocusing (Pentland, 1987). Although depth from defocus processes fewer images and has a faster-focusing speed (Gao and Ge, 2014), the focusing accuracy depends on the establishment of a correct focusing mathematical model (Rui, 2021), which can only be estimated theoretically at present, it is not completely accurate and just approach to idealization infinitely, which result in larger error effect (Meng, 2005). Depth from focus does not need to establish the mathematical model of the imaging system in advance, it is a method of focusing search process (Shiyun, 2022), whose core is focusing search algorithm and definition evaluation function (Yuhu et al., 2013). However, it still does not equip with good adaptability, and cannot get accurate definition evaluation on some collected images with multi-noise (Yu and Lu, 2022). Meanwhile, depth from focus algorithm needs to acquire and process a series of data that image from clear to fuzzy, which takes much time (Yipeng et al., 2005) and cannot satisfy both focusing accuracy and real-time at the same time, unable to coordinate the two to a favorable standard (Fan, 2021).

In recent years, with the rapid development of computer technology, deep learning has also ushered in explosive growth (Wang et al., 2016; Cao et al., 2021; Hu et al., 2022), and has achieved a good application prospect in computer vision tasks (Cao et al., 2022a) such as image classification (Cao et al., 2020; Cheng et al., 2021; Hussain et al., 2021; Safari et al., 2021; Hang et al., 2022), and object detection (Ranjan et al., 2018; Hassaballah et al., 2021; Cao et al., 2022b). By extracting the image deep feature information, and predicting information within a very short period, can greatly improve the validity and accuracy of the detection results. Therefore, the use of deep learning techniques for microscopic imaging autofocusing has become a focused research of biomedical microscopic images in recent years.

In 2018, Jiang et al. (2018) explored the application of deep convolution neural networks (CNNs) for microscope autofocusing. They used the trained model to predict the focal position of the acquired image without axial scanning, which significantly improved the autofocusing speed of the microscope and avoided the defects associated with autofocusing algorithm. In the following year, Pinkard et al. (2019b) designed a fully connected Fourier neural network based on coherent illumination, which uses an additional non-axial illumination source to predict the single image focus and emphasizes the generalization Capability between sample types. Dastidar (2019) First improved on input dataset by no longer acquiring multiple images in the vertical direction and maximizing the image sharpness to achieve autofocusing, instead, the difference image of two defocus images with a fixed spacing of 2 μm as inputs for deep convolution networks (CNN) to predict the optimal distance to be moved, to achieve the best focus relative for current position. In 2021, Luo proposed an autofocusing method (deep-R) based on deep learning. The network blindly and automatically outputs the focused image by training the sample microscopic image obtained at any defocus plane (Luo et al., 2020).

In the same year, Li et al. (2021) proposed a deep learning-based autofocusing framework that estimates the position of the focal plane of the objective lens relative to the plate by receiving two defocus images acquired by the fluorescence microscope of the plate, providing a deterministic measure in the prediction. Therefore, image blocks that may contain background or low-contrast objects can be excluded, improving accuracy. However, organisms have unique forms and characteristics, which may make microscopic images too different. The method proposed by Li needs to rely on a relatively large dataset to fit the ideal model, otherwise, the predictive performance of unseen samples will be reduced, the network generalization capability is weak, and the efficient prediction of multi-domain, multi-rate microscopic defocus images cannot be realized.

To accurately predict the focal length of defocus images, this paper proposes a deep learning network architecture with lightweight, faster computing speed, wider prediction area, and stronger generalization ability, while considering both efficiency and accuracy. The implementation of the method is described in detail from the construction of the dataset, model construction, and training method. A complete evaluation system is constructed, comparing and analyzing the performance gap of this network and other network models such as ResNet50 and BotNet. Finally, summarized and analyzed the important results of the experiment.

## 2. Construction of test dataset

### 2.1. Test facility and data acquisition

The dataset for this experiment consists of two parts, one using the open source dataset, and the other part was observed using the ML-31-M biomicroscope equipped with a 10X/22 large field of view eyepiece as standard (Provided by Guangzhou Mingmei Technology). Under the lighting conditions of the LED coherent illumination that comes with the device, an MD50-T microscope digital camera with 2.2 μm × 2.2 μm image element size and 5 megapixels was used to acquire an effective pixel high-resolution image of 2,592 × 1,944 size.

**Figure 1:**
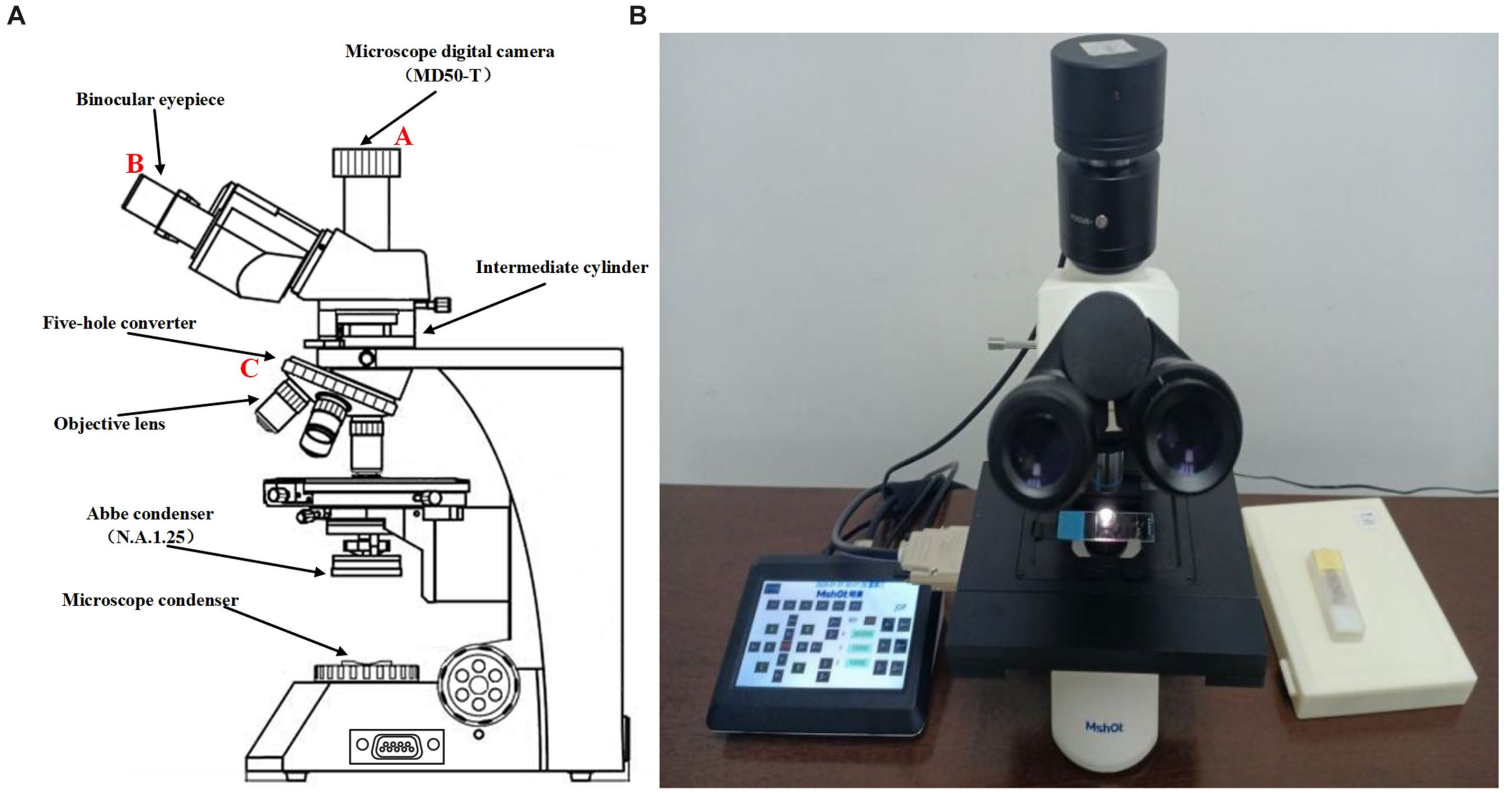
**(A)** Sketch of ML-31-M biological microscope. **(B)** Real picture of ML-31-M biological microscope.

**Figure 2:**
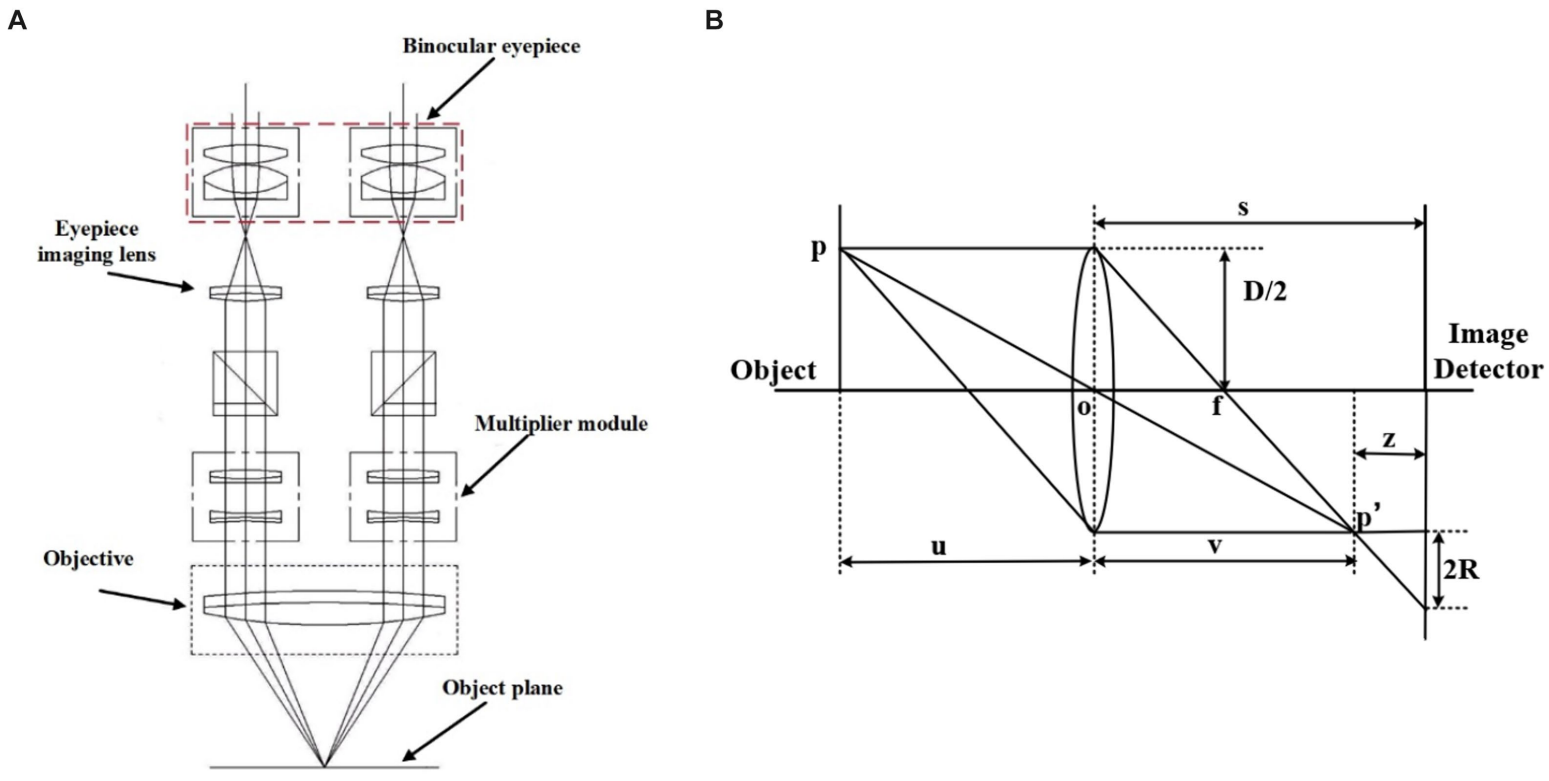
**(A,B)** are the principle diagram. **(A)** Binocular microscope imaging principle diagram (Wu et al., 2020). **(B)** Microscope imaging principle diagram.

Figure 1 shows the schematic diagram of the ML-31-M biological microscope, where part A is the MD50-T microscope camera with a resolution of 5 megapixels, which can provide a frame rate of 14fps in full pixel mode. Part B is an adjustable large field of view WF10X/22 mm double-headed eyepiece. C is a four-hole converter equipped with four infinity distance flat-field achromatic objectives of 10X/0.10, 20X/0.25, 40X/0.65, and 100X/1.25. The ML-31-M used is a binocular microscope with two fluoroscopic systems. The imaging principle is based on binocular stereo vision, where different parts of the objective are observed through different eyepieces and the images are subsequently combined through brain vision processes. As shown in Figure 2A, the sample slide forms an inverted real image by the magnification of the objective lens, and the light rays are secondarily magnified by the multiplier module, and then the light rays are cast down to the eyepiece imaging lens for convergence, and finally enter the eyepiece to form a magnified orthogonal virtual image to be observed.

The microscope imaging process is based on the imaging principle of the convex lens, and the schematic diagram is shown in Figure 2B. P is the observation point of the sample, s is the distance from the center of the convex lens to the image detector, D is the diameter of the lens aperture, and R is the radius of the blurred image point where p’ falls on the image detector. The relationship between focal length f, object distance u, and image distance v satisfies Gauss’s formula:


$$\frac{1}{u}+\frac{1}{v}=\frac{1}{f}\;\left(1\right)$$

When a clear flat image is observed, the viewing surface at this point is the focal plane of the system. But in defocus plane, will form a fuzzy image point on the observation surface, the radius of the image point R can characterize the degree of focus of the image, that the value of z in the figure is greater, the image is more away from the focal plane, the image point fuzzy circle is larger, the relationship holds:


$$\frac{R}{D/2}=\frac{z}{v}=s\left(\frac{1}{v}-\frac{1}{s}\right)\left(2\right)$$

From the above two equations, we can obtain:


$$R=s\frac{D}{2}\left(\frac{1}{f}-\frac{1}{u}-\frac{1}{s}\right)\left(3\right)$$

Clear imaging by changing the value of u\s\f so that the image plane is located at the focal plane.

**Figure 3:**
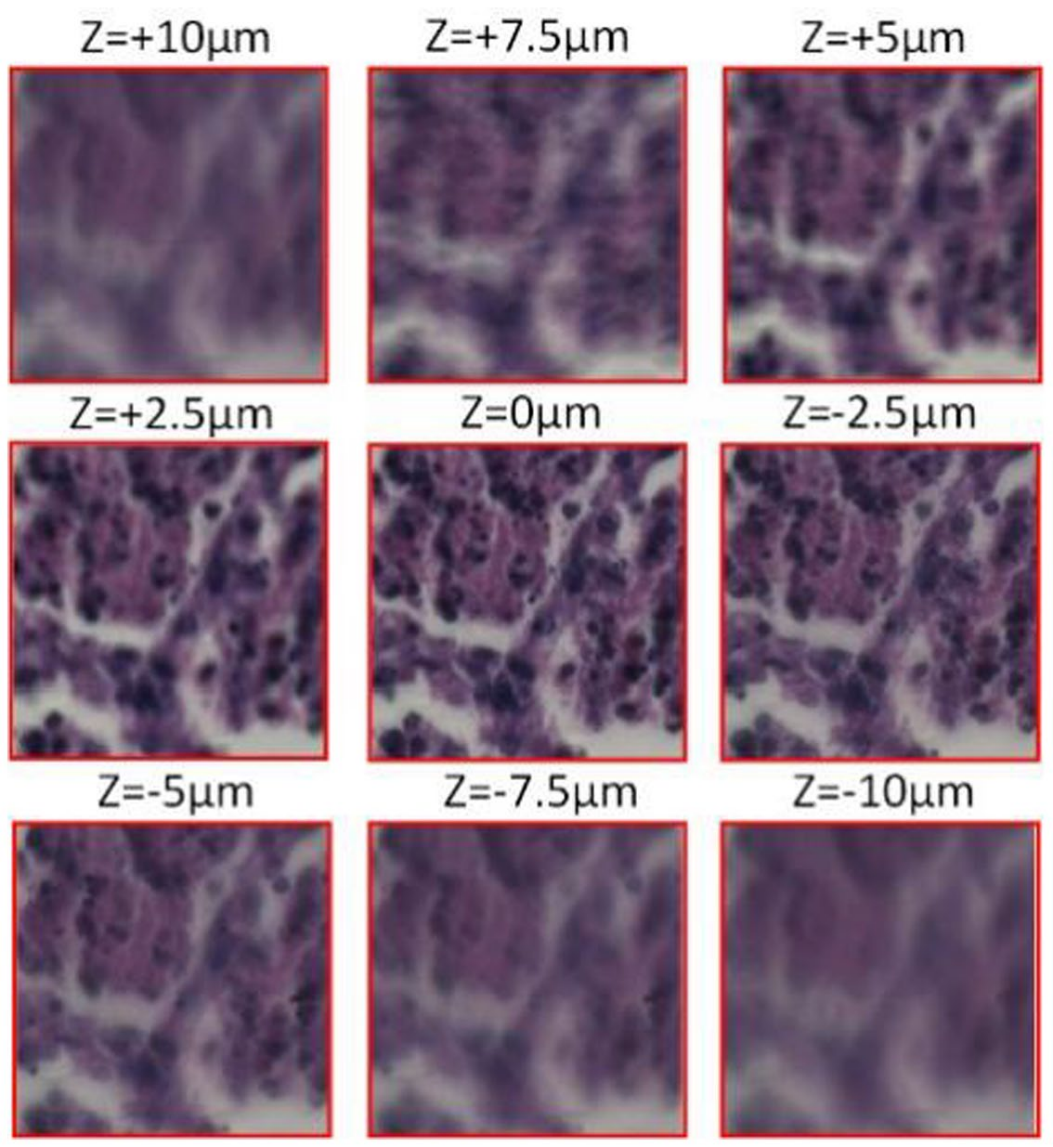
Cell micrograph (observed with a 20X Eclipse motorized microscope, acquired by Pointgrey BFS-U3-51S5C-C camera).

Figure 3 shows the microscopic imaging of the same centroid in the tumor cells depicting the imaging situation at different focal planes. When z = 0um, microscopic imaging is in the plane of focus when the image clarity is the highest. Subsequently, the defocus plane image is acquired by moving up and down a certain step, and the *z*-value is the distance the objective lens is moved with respect to the plane of focus. It is clear from the microscopic images that the further away from the defocus plane to the focal plane, the lower the sharpness of the image.

### 2.2. Building of the dataset

High-resolution microscopic images located in the focus can clearly observe the morphology and structure of the sample. However, it is impractical to achieve manual focusing in the face of a large number of data samples, so there is an urgent need to develop a method that enables accurate prediction of the focal length to achieve high-resolution autofocusing. To achieve the accuracy of the model, it is not enough that only use the public dataset. For this reason, the experiments in this paper use a two-part dataset.

**Figure 4:**
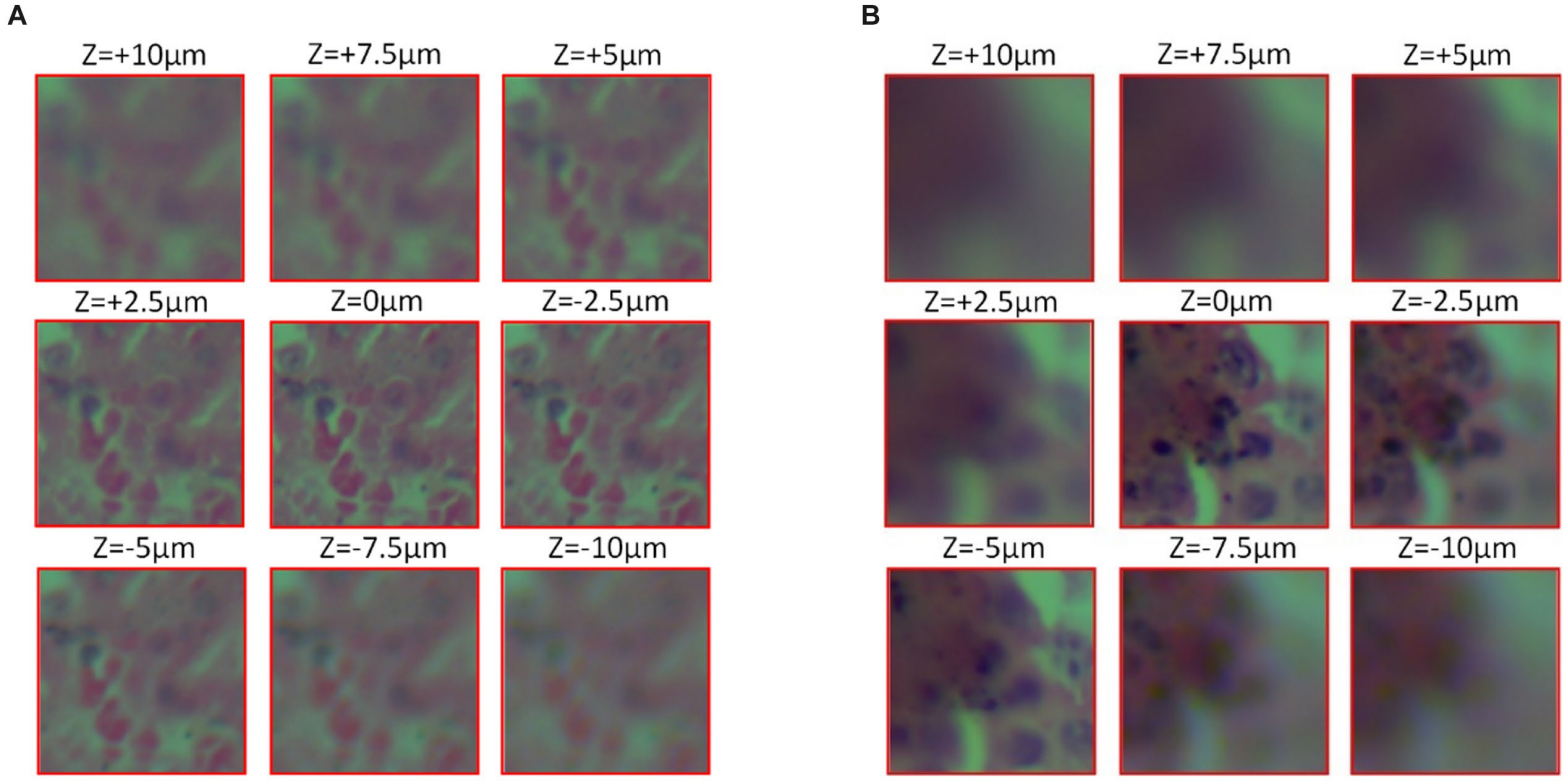
Cell micrograph (observed with the ML-31-M biological microscope, acquired by the MD50-T microscope camera). **(A)** 20X objective observation **(B)** 40x objective observation.

One part is a self-built dataset, that using the ML-31-M biomicroscope to collect. The process starts with the initial focusing of the sample, the next fine-tuning of the focus to achieve optimal definition, and move the sample to different defocus positions ranging from −10 μm to +10 μm in steps of 0.5 μm to obtain defocus images. As shown in Figure 4. The above steps were repeated for the entire sample in 1 mm lateral steps, and a total of 20 sets of data were collected, each containing approximately 40 images. Finally, the images and the corresponding focus position information were saved, and the defocus image under two magnifications of 20X and 40X were acquired by same method.

Another part is the public dataset (Jiang et al., 2018), micrographs were observed with an Eclipse electron microscope (provided by Nikon Eclipse) at 0.75 NA, 20X lens, which was obtained from a 5-megapixel color camera (Pointgrey BFS-U3-51S5C-C) with a 3.45 μm pixel size. Keeping the defocus distance range from +10 μm to −10 μm, 40 defocus stacks with 0.5 μm step spacing were captured in the same field of view, totaling 40 × 40 images in the same field of view, and the obtained images were segmented into approximately 130,000 images of size 224 × 224 for network training. This is shown in Figure 4.

## 3. Model structure

### 3.1. Method overview

Deep convolutional neural networks have been widely used in image classification and processing in recent years. In this paper, we use the collected defocus image data combined with convolutional neural networks to construct an end-to-end model to predict the focal length of an image, and maximize the requirements for high-accuracy prediction under multiple regions. The model is as follows:


$$D^{p}=F\left(S_{k,}\delta \right)\left(4\right)$$

$\;S_{k}\;$ denotes a 224 × 224 size defocus cell image, $D^{p}$ is the predicted focal length obtained after training the network model, and F is the regression function obtained after training, and δ is a set of network learning parameters including learning rate, number of iterations, etc. In the training process, by feeding a large number of dataset consisting of defocus images into the network, the training is continuously iterated to obtain the optimal parameters δ of the model, the gap between the predicted focal length $\;D^{p}$ and the real focal length $D^{t}$ is minimized, which makes the problem transformed into:


$$\delta_{m}=\arg m\mathrm{in}\;L_{\delta }\left(D^{t},\;\;D^{p}\right)\left(5\right)$$

For some wide-field high-resolution images, in order to get the focal length more accurately and quickly, it needs to be partitioned into small images of 224 × 224 in height and width for prediction respectively, and the results will be averaged so that the original model will be transformed into:


$$D^{p}=avg(\sigma \left(F\left(s^{1}{}_{k},\;\;\delta \right)+F\left(s^{2}{}_{k},\;\;\delta \right)+..+F\left(s^{h}{}_{k},\;\;\delta \right)\right)\left(6\right)$$

$D^{p}$ is the focal length of the predicted wide-field and high-resolution image, avg() is the averaging function, $s^{1}{}_{k},s^{2}{}_{k}\ldots$ is the same wide-field high-resolution image split into different 224 × 224 small images，and σ is the discriminant function, Because in the process of segmenting the wide-field high-resolution image into small images, a part of the image will include most of the blank area, resulting in unreliable prediction results obtained from this part of area, which needs to be discarded.

### 3.2. The proposed network structure

For the autofocusing of wide-field and high-resolution microscopic images, this paper proposes an LDSE-NET automatic focal distance prediction deep learning framework, using DenseNet as the main framework of the model in the network. Since 2015 He et al. (2016) proposed ResNet for the problems of vanishing gradient, explosion gradient and performance degeneracy that occur with deeper network layer structures, and the performance of deeper networks can be further improved by jumping connections between shallow and deep networks, weakening the strong connections between each layer. However, due to the large number of layers built by ResNet, more computational resources and time are required. So Huang et al. (2016) further improved the feature reuse capability based on ResNet and proposed DenseNet with dense connection operation.

**Figure 5:**
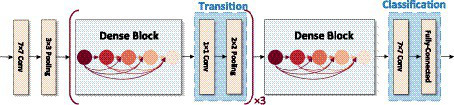
Network structure of DenseNet.

Figure 5 the input of each layer of the network and the output of all previous layers of the network, which mainly focuses on improving the network performance from the perspective of feature reuse, enhancing the feature propagation, and improving the efficiency of information and gradient transmission in the network. The network contains three layers structure of Dense block layer, Transition layer, and Classification layer. Where Dense block layer consists of a composite with BN, ReLU, and Conv nonlinear mapping functions, designed with a pre-activation strategy to make network training easier and generalization performance better; Conv represents the convolution layer in the deep neural network, which undertakes convolution calculation in the process of model reasoning. Transition layer is used for the connection between dense blocks and contains 1 × 1Conv, 2 × 2 Average pooling; Classification layer consists of Global average pooling and Fully connected layer, the input of each layer of the network includes the output of all previous layers of the network. Compared with ResNet50, this network mainly focuses on improving the network performance from the perspective of feature reuse, enhancing feature propagation, and improving the efficiency of information and gradient transmission in the network.

**Figure 6:**
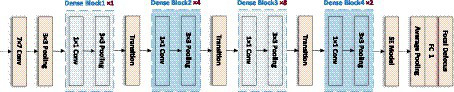
Network structure of LDSE-NET.

In addition, to emphasize the information feature channel, better adapt to the dataset, and further improve the prediction performance, the network architecture is adjusted and optimized in this paper. The number of Convolutional layers in the Dense Block is reduced, and some of the activation function in it are replaced with Tanh, making the network structure simpler and more efficient. On this basis, the SE module is connected after the last Dense Block to improve the accuracy of the image focal length prediction task to a certain extent. In this paper, the model is completed by sequentially superimposing the dense block, transition block, and squeeze excitation module. As shown in Figure 6.

The input of this CNN network structure is an unfocused blurred image captured by a microscope. The image is first passed through a Convolutional layer of a 7 × 7 matrix with a step size of 2 and a padding of 3 and then passed through a 3 × 3 maximum pooling layer with a step size of 2. The output is passed through the constructed Dense block layer in turn, compressing the Dense block layer input and all the extracted feature information with the help of Transition blocks, changing the size of the channels’ number so that the number of channels between adjacent dense blocks can correspond to each other, further enhancing the feature propagation between each layer, and the output is passed through the SE module to extract more feature information. Finally, the output is sent to the 7 × 7 Global average pooling layer and the Fully connected layer. The output of the network is a Regression layer, and the result is the predicted sample focal length.

#### 3.2.1. Dense block layer

Dense block layer is an important part of LDSE-NET, which is used to further improve the effectiveness of information transfer between each layer, and the specific propagation formula is as follows:


$$X_{L}=H_{L}\left(X_{0},X_{1},\ldots ,X_{L-1}\right)\left(7\right)$$

**Figure 7:**
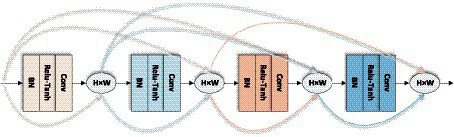
Dense block layer of LDSE-NET.

$\left[X_{0},X_{1},\ldots ,X_{L-1}\right]$ refers to the concatenation of the feature-maps produced in layer 0,1,…,L−1 , and $\left[H_{L}\right]$ is defined as a composite function of three consecutive operations consisting of normalization function, activation function, and convolution function, and the input of each layer is the output of the mapping results of all previous layers, and also the feature mapping result of the current layer is used as the input of the later layers, and the structure is shown in Figure 7.

#### 3.2.2. Transition layer

**Figure 8:**
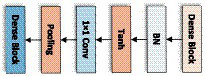
The transition layer of LDSE-NET.

The above-mentioned Dense block layer equation only works if the feature map is the same size, so the Transition layer needs to be used to do pooling and convolution to change the size of the feature map. So that the size of the feature map output from the Dense block layer is consistent with the shape size of the input of the next layer. The structure of the Transition layer used in this network is shown below, consisting of the BN layer of normalization function, Tanh of activation function, Conv of 1 × 1, and Average pooling of 2 × 2, and the structure is shown in Figure 8.

#### 3.2.3. Squeeze-excitation module

**Figure 9:**
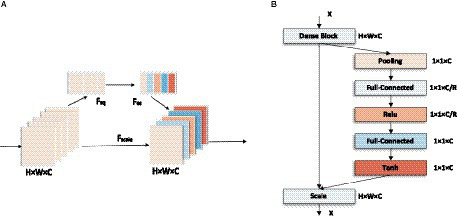
**(A)** Squeeze-Excitation module overall structure diagram. **(B)** Detail connection diagram.

The SE module improves the representativeness of the network by enabling it to perform dynamic channel-wise feature recalibration (Jie et al., 2018). This structure consists of Global average pooling layer, Fully connected layer, and linear activation function. The feature outputs of LDSE-NET are used as input to the SE module to increase the sensitivity to useful feature information. It learns the global information by fusing the convolutional features of each channel and filters out the less useful feature information to improve the expressiveness of the model. This is shown in Figure 9.

#### 3.2.4. Squeeze

Squeeze works by compressing the global spatial information into a single channel using Global average pooling. In principle, the channel statistics Z is achieved by reducing the spatial U dimension height and width, which can be summarized by the equation:


$$Z_{m}=F_{sq}\left(u_{m}\right)=\frac{1}{H\times W}\overset{H}{\underset{i=1}{\sum }}\overset{W}{\underset{j=1}{\sum }}u_{m}\left(i,j\right)\left(8\right)$$

#### 3.2.5. Excitation

This module is designed to take advantage of the global information obtained by compression and aims to fully capture channel-wise dependencies (Jie et al., 2018), and consists of two Fully connected layers and an activation function, as shown in Figure 9A. In order to better adapt to the data set of this experiment, the sigmoid activation function is changed to the Tanh activation function, so the excitation operation S can be summarized by the formula:


$$S=F_{es}\left(z,W\right)=\sigma \left(g\left(z,W\right)\right)=\sigma \left(W_{2}\delta \left(W_{1}z\right)\right)\left(9\right)$$

where $W_{1}\in R^{\frac{C}{r}\times C},W_{2}\in R^{C\times \frac{C}{r}},\delta \left(x\right)=\max\left(0,x\right)$ representing the ReLU activation function alleviates the vanishing gradient problem, and compared with Sigmoid activation function, $\sigma \left(x\right)=\frac{1-e^{-2x}}{1+e^{-2x}}$ improves the convergence speed, *z* is the channel information collected by the above Squeeze operation. The final output ${\tilde{X}}_{m}\in R^{H\times W}$ is obtained by multiplying the channels between the scalar $s_{m}$ and the feature map $u_{m}$ . This can be written as


$${\tilde{X}}_{m}=F_{scale}\left(u_{m},\;\;s_{m}\right)=s_{m}•u_{m}\left(10\right)$$

## 4. Experiment results and analysis

### 4.1. Model training

The experimental training process in this paper was run on a desktop computer with an NVIDIA GeForce RTX 3080 graphics card, an Inter Core i5-12600KF CPU and 32 GB of RAM. After some small sample tests, the parameters of the LDSE-NET were determined. Mean square error (MSE) was used as the model loss function, defined as:


$$Loss=MSE\left(D^{t},\;\;D^{p}\right)=\frac{1}{n}\overset{n}{\underset{i=1}{\sum }}{\left(D_{i}^{t}-D_{i}^{p}\right)}^{2}\left(11\right)$$

In the above equation, MSE() represents the root mean square error function, $D^{t}$ represents the true focal length in the dataset, $D^{p}$ represents the result predicted by the network, and n is the number of samples. The training optimizer uses Adam deep learning optimization algorithm, sets the network learning rate to 0.001, and uses the lr_scheduler mechanism to adjust the learning rate at certain epoch intervals to achieve a better training effect. The batch size is set to 50 images, and the training is stopped when the loss values of the test set and training set tend to stabilize and do not decline. Using RGB channels images from the public dataset, dividing the dataset RGB Channels images into training set and test set in a 9:1 ratio, and there is no intersection between them. To verify the performance of the network, this experiment compares the network structures of ResNet50 and BotNet (Bottleneck Transformer Network) and obtains the experimental results of each network structure separately.

### 4.2. Prediction results and analysis

**Figure 10:**
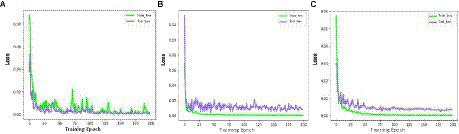
**(A–C)** represents train_loss and test_loss for ResNet50, BotNet, LDSE-NET three different networks.

According to the above indexes for training, the results of the prediction accuracy changes are shown in Figures 10A–C, the LDSE-NET has a small oscillation range of the loss values of the training set and the test set throughout the training process, and after about 50 epochs, the loss values of both the training set and the test set fluctuated within 0.005, and there was a significant decline in the training process, with the final model loss value stabilizing around 1E-05. On the contrary, the other two networks showed larger fluctuations in the loss values during the training process. The BotNet test set loss value fluctuated sharply between 0.01 and 0.02 and could not decrease; when ResNet50 had a sharp increase in error after training to a certain epoch, followed by a dramatically decrease, and the loss value could not be stable. The final model loss values of both networks can only drop to around 1E-04, and the training effect is poor compared to the LDSE-NET.

**Figure 11:**
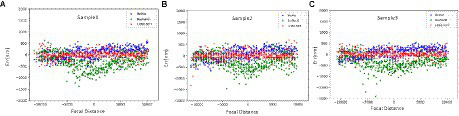
**(A–C)** Prediction error distribution of the three networks for sample 1, sample 2, and sample 3.

Besides, this paper compares three models in multiple sets of renal sample images of the focal length prediction results, and selects the error result distribution of three sets of data as shown in Figures 11A–C. From the figure, the three networks have little difference in the prediction effect in the range of −10 μ m to −5 μ m, because defocus images blur to a large degree and contain fewer image features, making each network have the same effect. In the interval of −5 μm ~ +5 μm, by evaluating the prediction error distribution, it can be found that most of the error distribution of LDSE-NET model is within +250 nm ~ −250 nm. In contrast, the prediction error of the ResNet50 model is mostly distributed beyond 500 nm. Overall, compared with BotNet and ResNet50 networks, the prediction accuracy of LDSE-NET network is improved by 1 and 2.5 times, respectively.

In addition to the improvement in prediction accuracy, the purpose of this paper is to increase the computational speed of the model as well as to make it more lightweight. The specific comparison results are shown in Table 1. All three networks were trained by the same hardware device, and when the model training was completed, the training time of LDSE-NET was about 6 h, and the speed was improved by 20% compared to BotNet and 33.3% compared to Resnet50. The model size is simpler and lighter than the other two networks, with only about 12% of the size of the two networks.

**Table 1:** Performance comparison of ResNet50, BotNet, and LDSE-NET.

NET	Focusing error (nm)	Model size	Final loss	Train time
ResNet50	0.5 ± 0.32	4.002 MB	3.89E-04	9 h
BotNet	0.38 ± 0.29	3.701 MB	2.04E-04	7.5 h
LDSE-NET	0.15 ± 0.17	480 KB	7.65E-05	6 h

### 4.3. Comparison of the predicted effect of variable magnification, variable area

To further evaluate the performance of the network. In this paper, we also use the 20 sets of data collected above containing a total of about 110,000 images of size 224 × 224 for training and testing, which are also divided into training set and test set in the ratio of 9:1, with no intersection between the two sets of data. The network models were trained according to the above-mentioned network parameter metrics to obtain the network models under 20X lens and 40X lens, respectively, and used to predict the focal length of defocus images under different magnifications.

**Figure 12:**
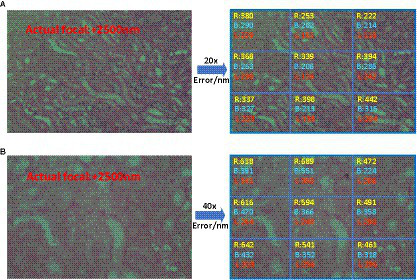
R, B, and L represent ResNet50, BotNet, and LDSE-NET three different networks. **(A)** Prediction error of 20X lens **(B)** Prediction error of 40X lens.

As shown in Figure 12, the predicted images were first divided into nine regions of 3 × 3, which do not have overlapping parts, and the focal length prediction was performed for these regions separately. Comparing the prediction results under the two magnifications, it can be seen that the prediction effect of 20X is better than 40X, and the error is reduced by about 100 nm ~ 200 nm. This is due to the fact that the field of view under the 40X lens is narrower and contains fewer cells, and the edge position becomes more blurred compared to the 20X lens, which makes each image may contain many blank areas after cutting into small images, resulting in its feature information is more blurred and sparse, which makes the prediction focal error increase. In addition, the prediction results of the network for the middle of the image are better than the edge locations, which is most likely because the entire field of view is too large for the microscope head and camera to focus over the entire field of view, resulting in the possible existence of more blurred locations on the edges. Therefore, during the training and prediction process, the image in the center of the field of view can be selected, and more accurate results will be obtained.

**Figure 13:**
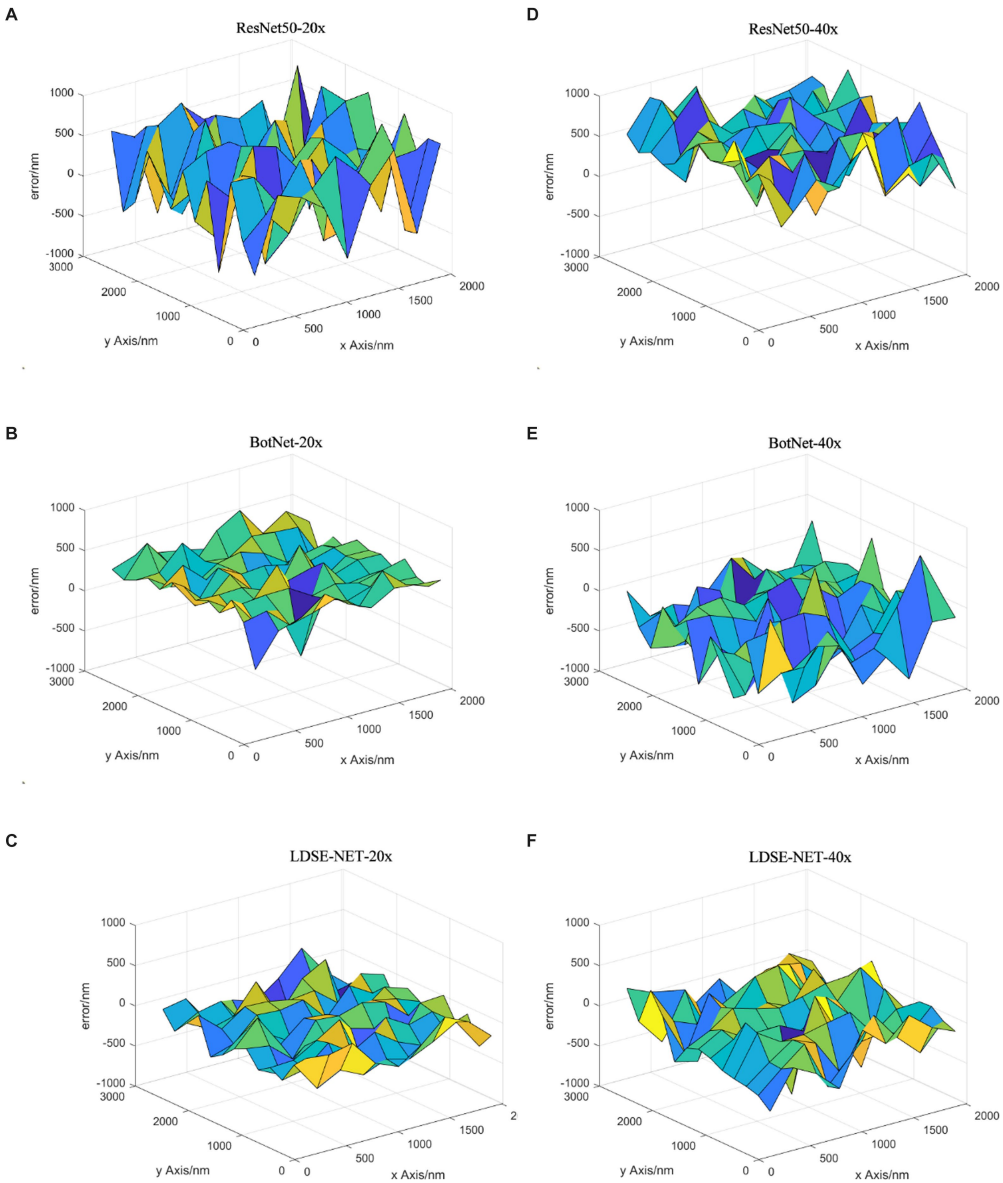
Focal length error of 20X, 40X images predicted by different networks.

Secondly, Figure 13 shows the prediction focal error plots of each of the three networks for the same high-resolution defocus image with a large field of view at different magnifications. Combining the results of this experiment, it can be seen that for 20X magnification images, the prediction error of ResNet50 and BotNet are mostly above 300 nm. On the contrary, most of the prediction error of LDSE-NET remain below 300 nm. Similarly, from the 40X magnification error map distribution, it can be seen that more than 60% of regions of ResNet50 and BotNet have error over 500 nm, while the average error of LDSE-NET is controlled around 300 nm. Therefore, the error of LDSE-NET is significantly smaller than the other two networks for both 20X and 40X magnification data, and the accuracy of some areas is improved by 1 ~ 2 times compared to BotNet and ResNet50.

**Figure 14:**
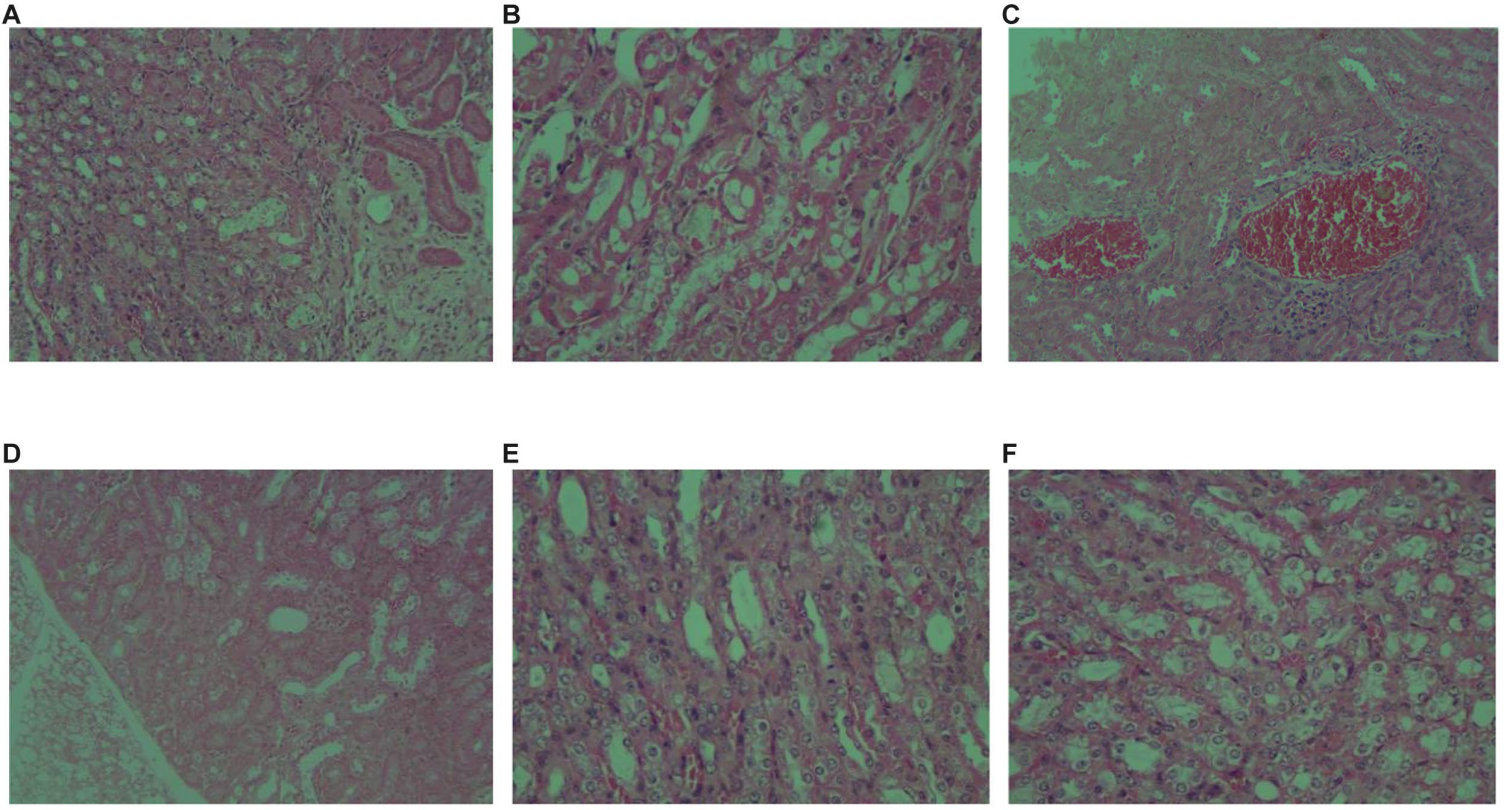
**(A–F)** represent picture1 ~ picture6 test sample images respective.

**Figure 15:**
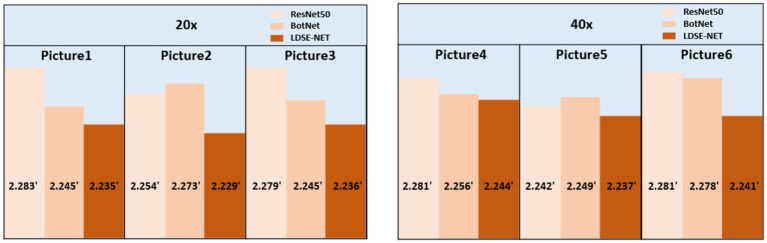
Comparison of the time required to predict a single image by different network predictions.

In addition, to satisfy the requirement for error reduction, the computational speedup is also an important purpose. Here, three models are utilized to directly predict the single image focal length, as shown in Figure 14, large-field high-resolution images of different regions of the same sample are selected, all experiments are conducted on the same computational platform and obtain the running time. The specific comparison effect is shown in Figure 15, in terms of time efficiency comparison, the computation time of LDSE-NET network is improved by 0.02 s ~ 0.04 s. Combined with the above experimental contents, this shows that this network is better than ResNet50 and BotNet in terms of accuracy and time.

## 5. Conclusion

In this paper, we present a dense model LDSE-NET with squeeze excitation for predicting the focal length of the defocus images under the medical microscope. Its effectiveness in focal length prediction is verified by using multi-region and multi-magnification image data. Through the evaluation of the prediction results in the test set, compared with the other two networks of BotNet and ResNet50, the accuracy of the image focal length prediction of LDSE-NET is improved and the model proposed is lighter. This network reduces the information loss, improves the transmission efficiency of information in the network, and further proves the feasibility and practicability of deep learning in the prediction of focal length of microscopic imaging on the basis of previous studies, and provides ideas for future research.

## Data availability statement

The original contributions presented in the study are included in the article/supplementary material, further inquiries can be directed to the corresponding author.

## Author contributions

All authors listed have made a substantial, direct, and intellectual contribution to the work and approved it for publication.

## Funding

This study was supported by Natural Science Foundation of Sichuan Province, Grant/Award Number: 23NSFSC1257.

## Conflict of interest

The authors declare that the research was conducted in the absence of any commercial or financial relationships that could be construed as a potential conflict of interest.

## Publisher’s note

All claims expressed in this article are solely those of the authors and do not necessarily represent those of their affiliated organizations, or those of the publisher, the editors and the reviewers. Any product that may be evaluated in this article, or claim that may be made by its manufacturer, is not guaranteed or endorsed by the publisher.
